# Decision-making Ability of Professional Managers Based on Neurocognitive

**DOI:** 10.1515/tnsci-2019-0022

**Published:** 2019-05-31

**Authors:** Guo Liang, Xiyuan Li

**Affiliations:** 1 School of Business, Zhejiang Wanli University, Ningbo, Zhejiang 315100, China; 2 School of Economics and Management, Wuhan University, Wuhan, Hubei 430072, China

**Keywords:** Neurocognitive science, professional managers, decision-making power, influencing factors

## Abstract

In order to explore the factors influencing managers' decision-making ability and the relationship among them, this study puts forward the hypothesis on the basis of analysing the factors influencing the decision-making ability, constructs a neurocognitive model of the factors influencing the decision-making ability, analyses and verifies the hypothesis with the neurocognitive model in combination with the questionnaires, and calculates the path coefficient through the model. The empirical results show that decision-making information, leadership authority and corporate culture have significant influence on the decision-making ability, in which leadership authority is the main factor, and governance structure and decision-making system indirectly influence the decision-making ability through other intermediaries.

## 1 Introduction

Work design and motivation were initially seen as two different management functions, and managers rarely linked the two. In the era of knowledge economy, human capital has become one of the most important capitals for enterprises in the fierce market competition. Work design has begun to play a pivotal role in the issue of employee motivation. In addition to setting a reasonable salary system and motivating employees’ external work motivation through material rewards, the company began to stimulate employees’ internal motivation through work design. Although scholars have proposed work feature models, the theory of work design is more from the experience summary in management practice, less theoretical discussion on the basic psychological needs of employees, and the self-determination theory of motivation questions can provide important theory for work design. Explain and guide. According to this theory, individuals generally have three basic psychological needs: autonomy, competence, and belonging. Among them, the degree to which autonomy and competence need to be satisfied will have an important impact on the level of intrinsic motivation of individuals.

Decision-making is a universal phenomenon in real life and an important part of management field. As an important part of management science, decision-making science has drawn attention from other subjects besides management science, such as economics, psychology, and cognitive neuroscience [1]. Decision-making neuroscience is a very new frontier discipline and a new growing point of management science. It is a cross-discipline developed on the basis of the traditional behavioural decision-making science and tries to use the imaging technology of neuroscience. In combination with the recent progress of cognitive science and psychology, and the decision-making science of management science, it studies the processing mechanism behind human decision-making behaviour, and open up the “black box” for decision processing of the brain [2].

By interpreting the processing process of decision-making at the brain level and understanding the mechanism of decision-making process at the level of neuroscience, the study re-examines the traditional behavioural decision-making model to provide empirical evidence for remoulding the theoretical framework and normative basis of decision-making model. Based on neurocognitive science, this paper has carried out a series of studies on the decision-making ability of professional managers, and analysed and summarized the various factors that influence the decision-making ability.

Based on the factors affecting managers’ decision-making ability and their interrelationships, this paper constructs a neural cognitive model that influences the factors of decision-making. The neurocognitive model is combined with the questionnaire production ability, the hypothesis is analysed and verified, and the path coefficients are calculated by the model. The empirical results show that decision-making information, leadership authority and corporate culture have a significant impact on decision-making ability.

## 2 The application of neurocognitive science in decision-making mechanism

### 2.1 The core of the decision

**Figure 1:**
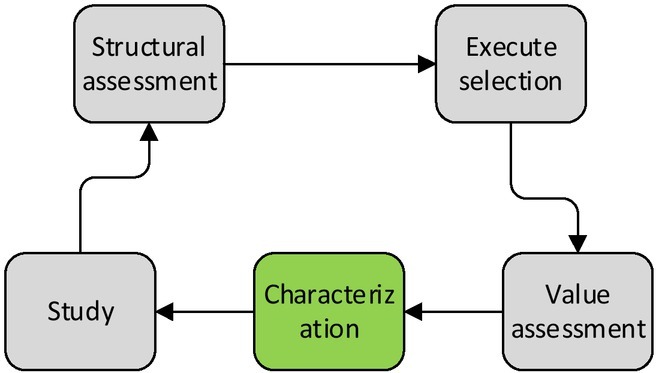
Decision flow chart

The decision-making of organisms, including human beings, is a dynamic and continuous process, which is divided into five steps, that’s, characterization, value assessment, action selection, result evaluation and learning, and it is a complete decision from characterization to result evaluation, while learning by evaluating the results of current rounds of behaviour is to provide new information for the next behaviour selection, and make continuous dynamic adjustments. The characterization mainly refers to the identification of the internal and external states by individuals before making the decision, so the decision-making closely related to the decision-makers involves three stages, namely, value evaluation, execution selection and result evaluation, then learning to enter the next decision-making, with a complete decision-making process as shown in Figure 1 ^[3]^.

### 2.2 Neural decision theory

**Figure 2:**
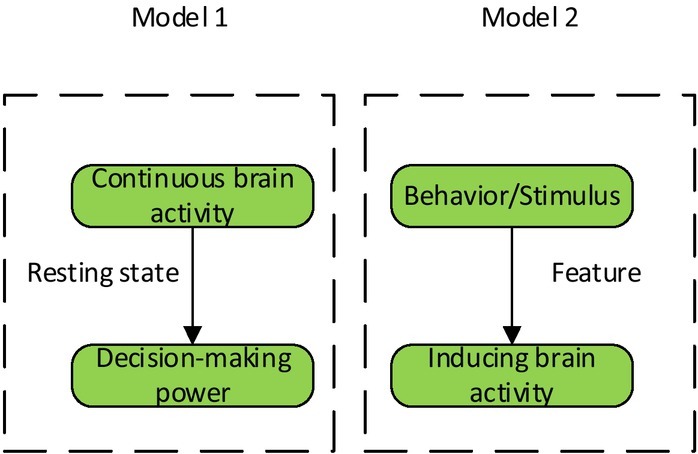
The causal model of decision-making behaviour and brain activity

The emergence of neuroscience extends the theoretical framework of decision-making ability. Two causal models constructed by Waldman provide effective solutions to the above disputes (as shown in Figure 2). Model 1 represents “brain activity inducing decision-making behaviour”, which is based on the theory that the brain has a relatively stable neural structure, which is capable of inducing certain decision-making behaviour and can be evaluated in a resting state of the individual. Model 2 indicates that “behaviour/stimulation inducing brain activity,” i.e., the decision-maker’s behaviour or stimulation in a particular experimental scenario can induce brain activity. The model takes decision-making behaviour or related stimuli as independent variables, and takes the activation of corresponding brain regions as dependent variables. This type of study uses neural techniques to record brain or neuroimaging when subjects receive visual or acoustic stimuli or operational tasks. Finally, differences in brain structure or brain activation are used to explain the mechanism of decision-making behaviour ^[4]^.

For both models, we are not going to discuss what is right or wrong. In fact, the two models complement each other, that’s, they provide a theoretical framework for revealing and enriching the mechanism of decision-making ability and brain activity from different research ideas and operating mechanisms. Therefore, in future research, we should combine these two models, consider not only the mechanism of interpreting brain activity and predicting decision-making behaviour, but also explore the brain nerve mechanism of decision-making behaviour, in order to better uncover the brain mechanism of decision-making ability.

### 2.3 Neural imaging based decision making process observation method

**Figure 3:**
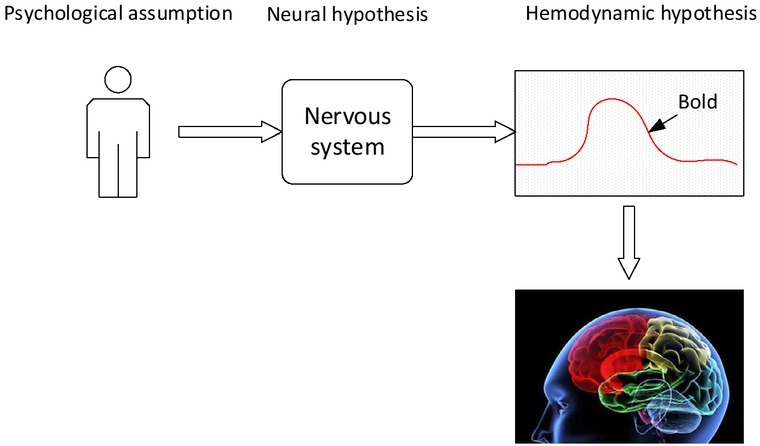
Neuroimaging reflects the mechanism of the decision-making process

The main techniques used in cognitive neuroscience experiments include functional magnetic resonance imaging (FMRI) and event-related potentials (ERP). Both techniques have their own advantages. The FMRI technology has very high spatial accuracy and can accurately observe the activation of the relevant brain regions in the brain in the main cognitive processing stage of the experimental process. ERP technology has extremely high time accuracy of the millisecond level, and can examine the brain’s cognitive processing process through the electrophysiological signals of the brain’s scalp surface at various stages of the individual task execution process (including the duration of only a few hundred milliseconds). The implementation method is shown in Figure 3 ^[5]^.

## 3 Analysis of factors affecting decision-making power

The decision-making ability of professional managers is related to the success of enterprises, and is influenced by individual factors, organizational factors and external environmental factors. This study analyses the influence of governance structure, decision-making system, decision-making information, and leadership authority and enterprise culture on the decision-making ability of professional managers.

### 3.1 Governance structure

When the external environment of an enterprise changes, the enterprise adjusts its own decision-making structure and decision-making mechanism in a timely manner, and a good corporate governance structure will be beneficial and can help the enterprise recognize the environment and the enterprise’s own capabilities and select the appropriate and competent management team, thus continuously improving the decision-making ability of the enterprise, and finally making the enterprise have the competitive advantage and long-term development ability.

A good corporate governance structure must have reasonable equity structure, board structure, board of supervisors’ mechanism and manager incentive mechanism. The equity structure is the most important factor in corporate governance structure, and determines the principal-agent relationship between managers and owners. The manager incentive mechanism can promote the agent’s behaviour goal keep consistent with the goal that the principal want to achieve to the greatest extent [6].

### 3.2 Decision system

Decision-making system refers to the organizational system formed by the administrative decision-making organization and personnel and the relevant system of decision-making, and has an important influence on the decision-making ability of enterprise leaders. Especially when the enterprise is in a bad situation or faces an unexpected incident, the advantages and disadvantages of decision-making system directly affect the decision-making ability of enterprise leaders. When evaluating and comparing the decision-making systems of enterprises, three criteria such as the decision-making error rate, decision-making correction rate and decision-making adaptability can be used to evaluate. The error of decision-making is divided into positive error and negative error. The positive error indicates the actual result achieved by the enterprise is greater than the expected result. The negative error indicates that the decision-making of the enterprise is not accurate. The decision-making correction rate is related to many factors, the low correction rate does not mean the decision-making quality is high, and the correction rate may be reduced by the factors such as information obstruction and system rigidity. In addition, the high correction rate does not mean the low decision-making quality, and may indicate that the enterprise is more adaptable to the complex and changeable external environment.

### 3.3 Decision information

Decision-making information is essential in decision-making process. Decision-makers must rely on effective decision-making information to make decision-making. Scientific decision-making not only needs complete and reliable information and reasonable and effective analysis method, but also needs to correctly understand and grasp the information and its characteristic essence. The influence of decision-making information on decision-making ability is mainly reflected in the timeliness, authenticity, quantity and repeatability of decision-making information, as well as the cost and benefit of decision-making information.

### 3.4 Leadership authority

Leadership authority is the unification of position power and personal charm, and influences the decision-making ability of enterprise leaders. Too high or too low position will result in lower decision-making ability. Too high position power may result in dictatorship and autocracy, which leads to the lack of flexibility of leadership system, bondage of subordinates, and the attack of enthusiasm, initiative and creativity, reducing decision-making ability. Too low position power can lead to difficulties in unification, weakening each other, wasting resources and reducing decision-making ability. In addition, when a leader has charisma, he or she can depend on his or her influence to influence his or her subordinates.

### 3.5 Company culture

When corporate decision-making conforms to the value orientation of corporate culture, it will be implemented more efficiently and smoothly. The influence of corporate culture on the implementation of decision-making is to produce psychological identity and shape common vision. Psychological identification will make the cultural atmosphere of the enterprise more improved and consolidated, thus forming a virtuous circle and interaction and improving decision-making ability. The common vision can generate the group psychological impetus and pressure for the conformity of individual behaviour, and then make the enterprise members form the psychological resonance and restriction, so as to achieve the self-control and restriction of the enterprise members to their behaviour, thereby improving the decision-making ability ^[7]^.

## 4 The establishment of a decision model and empirical research

### 4.1 Decision model

Neurocognitive model is a method to model multiple variables and test specific hypotheses simultaneously. Because decision-making ability of professional managers is the result of multiple factors, different factors have different influence on decision-making ability of professional managers. In order to define the influence degree of each factor on decision-making ability of professional managers and identify the key influence factors, neurocognitive models can be used ^[8]^.

**Figure 4:**
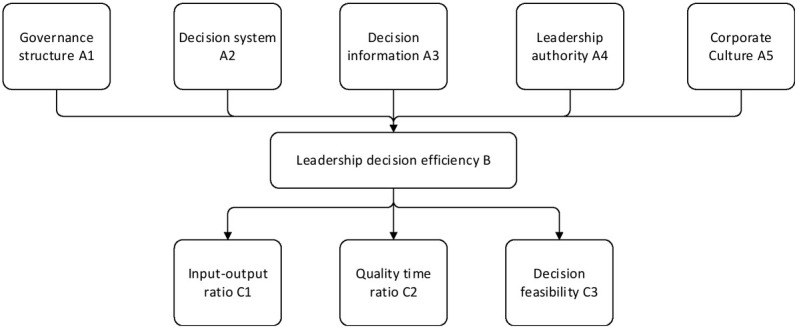
Professional manager’s decision-making concept model

The neurocognitive model is used mainly because it has the advantages of dealing with multiple variables, revealing the relationship among variables and the degree of influence simultaneously. In addition, the neurocognitive model can verify the influence of each factor and identify the key factors through sample data ^[9]^. By analysing the influence of governance structure, decision-making system, decision-making information, leadership authority and corporate culture on the decision-making ability of professional managers, a neurocognitive model is constructed, as shown in Figure 4.

There are 5 exogenous latent variables in Figure 4: governance structure, decision-making system, decision-making information, leadership authority and corporate culture; and 1 endogenous latent variable: decision-making efficiency ^[10]^. The variable table is shown in Table 1.

**Table 1:** Table of neurocognitive model variables

Latent variable	Observing indicators
Decision efficiency	Professional managers can make high output with low input
	Professional managers can make high quality decisions in a short period of time
	The feasibility of professional managers’ decision-making is high
Governance structure	Equity structure will affect professional manager’s decision-making efficiency
	Board structure will affect the efficiency of professional managers
	Supervisory Board Mechanism Affects Decision-Making Efficiency of Professional Managers
	Manager’s incentive mechanism will affect professional manager’s decision-making efficiency
Decision system	The miscalculation of professional managers will reduce the efficiency of professional managers’ decision-making
	Professional managers’ decision-making changes will affect professional managers’ decision-making efficiency
	The adaptability of decision-making of professional managers will affect the efficiency of decision-making of professional managers
Decision information	The timeliness of decision-making information will affect professional managers’ decision-making efficiency
	The authenticity of decision information will improve professional manager’s decision-making efficiency
	The amount of decision-making information will affect professional managers’ decision-making efficiency
	Information gathering costs and benefits can affect professional managers’ decision-making efficiency
Leadership authority	Professional manager’s position power will affect professional manager’s decision-making efficiency
	The Personal Charm of Professional Managers Improves the Efficiency of Professional Managers’ Decision-making
Company culture	Employees’ psychological identification with enterprises will improve professional manager’s decision-making efficiency
	Corporate employees have a common vision to increase the efficiency of decision-making for professional managers

### 4.2 Empirical research

The data are collected by means of questionnaire, which is designed with the scale of Likert5 (5 represents strongly agree, 4 represents relatively agree, 3 represents disagree, 2 represents relatively disagree, and 1 represents strongly disagreed), which reflects the influence of the above-mentioned five factors on the decision-making ability of enterprise managers.

The survey samples are mainly from the employees of Company A. 350 copies of paper and electronic questionnaires are issued by means of on-the-spot interview and mail delivery, 278 copies are collected, deducting 78 copies which are unqualified, and finally, 200 copies of valid questionnaires are obtained, with a valid collection rate of 57.14%.

Through the statistical analysis of 200 valid questionnaires, the subjects’ gender, age, education level, working years and jobs were mainly investigated. These data are more evenly distributed, and the recovery rate of the questionnaire reaches 95%. The data is valid and reasonable. The specific statistical results are shown in Table 2.

**Table 2:** Demographic characteristics of the respondents (N=200)

		Quantity	Percentage
Gender	Male	110	55%
	Female	90	45%
Age	Under 22	26	13%
	22-30 years old	49	25%
	31-40 years old	57	29%
	40+	68	34%
Education level	Specialist	19	10%
	Undergraduate	36	18%
	Master’s degree	78	39%
	Doctor and above	67	34%
Working years	1-3 years	25	13%
	3-5 years	42	21%
	5-10 years	31	16%
	More than 10 years	102	51%
Work position	General staff	58	29%
	Grassroots management staff	20	10%
	Middle management	45	23%
	Senior management	77	39%

**Figure 5:**
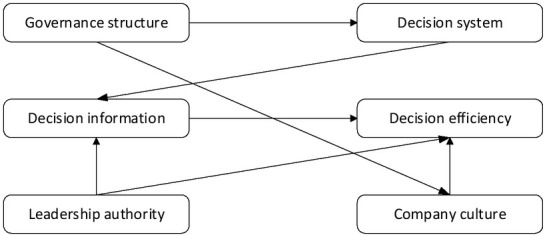
Decision-making path map

The fitting degree of the decision-making ability model is checked by using AMOS 17 1.0, and the path coefficient is shown in Figure 5, which shows that decision-making information, leadership authority and corporate culture have a significant impact on the decision-making efficiency of managers, and have a significantly direct impact on them. The governance structure has no direct influence on the decision-making ability of enterprise managers, but it indirectly influences the decision-making ability of enterprise managers through decision-making system and decision-making information, leadership authority and corporate culture.

## 5 Conclusions

The study of cognitive neuroscience can not only help us explore the essence of cognitive phenomena, explore the relationship between matter and consciousness, solve ancient philosophical problems, but also help us understand some phenomena in real society and improve the quality of our lives. The results of cognitive neuroscience can directly serve society. For example, a brain-damaged patient can perform a brain function imaging test to determine the brain area in which he is responsible for important cognitive functions (such as language) before surgery. Neurosurgeons can avoid damage to these brain regions during surgery. It is no exaggeration to say that cognitive neuroscience has penetrated into every aspect of our lives.

Based on the neurocognitive science, this study constructs the decision-making ability model. From the governance structure, decision-making system, decision-making information, leadership authority and enterprise culture, this study defines the influence degree of each factor on the decision-making ability of enterprise managers and identify the key influencing factors.

The main conclusions are as follows: decision-making information, leadership authority and corporate culture have significant influence on decision-making ability. Among them, leadership authority is the main influence factor, and governance structure and decision-making system indirectly influence decision-making ability through other intermediaries.
